# Validation of nocturnal resting heart rate and heart rate variability in consumer wearables

**DOI:** 10.14814/phy2.70527

**Published:** 2025-08-20

**Authors:** Michael B. Dial, Margaret E. Hollander, Emaly A. Vatne, Angela M. Emerson, Nathan A. Edwards, Joshua A. Hagen

**Affiliations:** ^1^ Human Performance Collaborative, Office of Research The Ohio State University Columbus Ohio USA; ^2^ STRONG Lab 711th Human Performance Wing, Air Force Research Laboratory, Wright‐Patterson Air Force Base Dayton Ohio USA

**Keywords:** consumer wearables, health monitoring, heart rate variability, sleep monitoring, wearable technology

## Abstract

Modern wearable devices report several heart rate‐based nocturnal health metrics, including resting heart rate (RHR) and heart rate variability (HRV). The purpose of this study was to assess the validity of nocturnal RHR and HRV from five wearable devices (Garmin Fenix 6, Oura Generation 3, Oura Generation 4, Polar Grit X Pro, & Whoop 4.0) against an electrocardiogram (ECG) reference. Thirteen healthy adults (6 females) wore an ECG reference and multiple wearables simultaneously during sleep, totaling 536 nights. Interdevice accuracy varied significantly (*p* < 0.05). For RHR, Oura Gen 3 (Lin's Concordance [CCC] = 0.97, mean absolute percentage error [MAPE] = 1.67 ± 1.54%) and Gen 4 (CCC = 0.98, MAPE = 1.94 ± 2.51%) demonstrated the highest accuracy, outperforming Polar's poor (CCC = 0.86, MAPE = 2.71 ± 2.75%) and WHOOP's moderate agreement (CCC = 0.91, MAPE = 3.00 ± 2.15%). Garmin was excluded from RHR analyses due to methodological inconsistencies. For HRV, Oura devices provided the highest accuracy; Oura Gen 4 (CCC = 0.99, MAPE = 5.96 ± 5.12%), Oura Gen 3 (CCC = 0.97, MAPE = 7.15 ± 5.48%). WHOOP showed moderate accuracy (CCC = 0.94, MAPE = 8.17 ± 10.49%), followed by poor agreement from both Garmin (CCC = 0.87, MAPE = 10.52 ± 8.63%) and Polar (CCC = 0.82, MAPE = 16.32 ± 24.39%). Oura devices showed the highest agreement for RHR and HRV, and WHOOP showed acceptable agreement, whereas Garmin Fenix and Polar demonstrated lower concordance, highlighting the importance of continuous validation and providing valuable benchmarks for clinicians, researchers, and consumers.

## 1 INTRODUCTION

Commercial wearable devices have become increasingly popular and useful for continuous monitoring of health metrics in recent years (Liguori et al., 2018; Newsome et al., 2024). For the last decade, wearable technology has been within the top three Worldwide Fitness trends identified by the American College of Sports Medicine (Newsome et al., 2024). These devices provide metrics pertinent to health, such as physical activity level (step count), energy expenditure (calories), and heart rate. Heart rate (HR) sensors embedded within watches, rings, smartphones, and wristbands provide non‐invasive avenues to provide useful information about cardiovascular function. While traditional clinical methods like electrocardiography (ECG) remain gold‐standard diagnostic tools, their practicality is limited by invasiveness, cost, and the inability to offer continuous monitoring outside clinical settings. Wearable technologies bridge this gap by enabling long‐term, continuous, and remote monitoring, offering clinicians actionable insights into patient populations, such as those with cardiovascular disease (Bayoumy et al., 2021; Singhal & Cowie, 2020), chronic stress (Hickey et al., 2021), and sleep disorders (Shelgikar et al., 2016), or elderly individuals requiring constant monitoring. As data generated by commercial wearable devices may potentially be used to report the health status of patients and help in the decision‐making process concerning patient prognoses and treatment options (Mizuno et al., 2021; Prieto‐Avalos et al., 2022), ensuring the validity of such metrics from these devices is critical.

Most wearable devices that measure HR use photoplethysmography (PPG) to measure acute changes in peripheral arterial blood volume, which reflect cardiac‐induced pulsatile flow in small arteries and arterioles (Allen, 2007). In short, PPG sensors are equipped with light emitting diodes (LED) and a photodetector. Light emitted from the LEDs penetrates the skin and capillaries, where light is then reflected to the photodetector. Green LEDs are normally utilized in wearables, as these wavelengths (500–570 nm) are absorbed into oxygenated hemoglobin, where the photodetector in the wearable device can then monitor the changes in capillary blood perfusion with each heartbeat and thus detect HR (Fukushima et al., 2012). PPG has previously been shown to be a reliable method to capture HR data compared to electrocardiography (ECG) in young and otherwise healthy populations (Maeda et al., 2011; Rehman et al., 2024) as well as those with cardiovascular conditions (Avram et al., 2019; Blok et al., 2021; Wouters et al., 2025).

PPG technology, despite its potential for continuous monitoring, is highly sensitive to noise, especially during movement, which can affect the accuracy of derived cardiovascular metrics (Ismail et al., 2021). For this reason, it is crucial that devices are worn appropriately on the peripheral wrist or finger according to manufacturer recommendations. While normal daily activities and exercise may reduce the sensitivity of PPG data, it is well validated during resting conditions and sleep (Biswas et al., 2019; Fukushima et al., 2012; Pradhan et al., 2019). Cardiovascular measurements, such as resting heart rate (RHR) and heart rate variability (HRV), are important parameters for evaluating sleep quality and overall health. Hence, evaluating the quality of the signal producing these reliable metrics through continuous monitoring is critical.

Valuable information about the body's health and recovery status may be available at rest and during sleep through cardiac measures. RHR is an independent predictor of both cardiovascular and all‐cause mortality regardless of a cardiovascular disease diagnosis (Fox et al., 2007; Palatini, 2007). Chronically elevated RHR during sleep is associated with cardiovascular morbidity and mortality, with elevated risk similar to factors such as smoking, hypertension, and dyslipidemia (Cook et al., 2006). Conversely, lowered RHR is associated with decreased all‐cause mortality and higher cardiorespiratory fitness (Cook et al., 2006; Gonzales et al., 2023; Jurca et al., 2005; Reimers et al., 2018). HRV is the variation in time between consecutive heartbeats and is a useful metric for determining the overall state of the autonomic nervous system (Shaffer & Ginsberg, 2017; The et al., 2020). HRV has been demonstrated to be a predictor of mortality following cardiovascular events (Guzzetti et al., 2005; Song et al., 2014). While both metrics are indicators of overall health and wellness, they are also very good indicators of day‐to‐day response to physiological stress stemming from training (Hall et al., 2004; Nuuttila et al., 2022). Both RHR and HRV are now widely reported by most wearable devices and their corresponding smartphone applications. When used correctly, longitudinal monitoring of RHR and HRV can be useful in healthy populations to identify trends, track recovery, and potentially deliver highly personalized feedback.

While many devices utilize the same PPG technologies for monitoring biological signals like RHR and HRV, each device implements proprietary algorithms that directly impact signal acquisition, filtering/cleaning, and computing of final metrics. The devices and algorithms also differ in the frequency of PPG data collection and the duration of the collection period, and others weight HR data collected during early or late‐stage sleep (WHOOP, n.d.; Garmin Ltd, 2025a; Oura Inc, 2024; Polar Electro Oy, 2024). As wearable devices become increasingly common, directly comparing outputs from multiple devices is useful both to quantify discrepancies and to evaluate practicality.

As RHR and HRV are important physiological vital signs that provide insight into general health, recovery, and sleep performance, it is vital to assess the validity and reliability of the wearable sensors reporting these data to the consumer. Most importantly, with the speed of innovation in the commercial wearables sector, hardware, software, and algorithms continue to improve and must be continuously assessed. Thus, the objective of the present study was to investigate the validity of sleep‐based physiological metrics, commonly measured by consumer wearable devices, specifically RHR and HRV.

## 2 METHODS

### 2.1 Participants

Participants were recruited via word of mouth among contractors and civilians at Wright‐Patterson Air Force Base (Ohio, USA), as well as university students and staff at The Ohio State University. Inclusion criteria included being over 18 years of age and not having any medical conditions or symptoms that chronically impaired sleep. All participants were instructed and informed about the study procedure and purpose, given the opportunity to ask questions, and each provided verbal consent to testing and participation. This verbal consent was witnessed in person and documented digitally by the researchers. This study was approved by the Institutional Review Board of the Air Force Research Laboratory (protocol; FWR202200227N), and was compliant with the Declaration of Helsinki guidelines (World Medical Association, 2013).

### 2.2 Devices

#### 2.2.1 Polar H10

The Polar H10 was used as the criterion measurement for heart rate (Polar, Kempele, Finland). The Polar H10 chest strap uses single‐lead ECG to measure heart rate while secured around the chest and has a sampling frequency of 1000 Hz (Polar Electro, 2019). The Polar H10 device has been studied thoroughly and shown to be valid and reliable for obtaining heart rate and R‐R interval data compared to ECG at rest and during exercise (Gilgen‐Ammann et al., 2019; Skala et al., 2022; Speer et al., 2020). Additionally, the H10 has a highly user‐friendly form factor and data capture features, including a comfortable, adjustable elastic strap that helps maintain skin contact (promoting high‐quality data collection) without becoming too uncomfortable to impair sleep.

#### 2.2.2 Wearable devices

Five consumer wearable devices were utilized to assess the accuracy and validity of sleep physiology measures. Three devices were wrist‐based devices, including the Garmin Fenix 6 (Garmin, Olathe, KS, USA), Polar Grit X Pro (Polar, Kempele, Finland), and the WHOOP 4.0 (WHOOP, Boston, MA, USA), and two devices were ring‐based, the Oura Generation 3 and Oura Generation 4 (Oura, Oulu, Finland). These devices were selected for analysis for various reasons, including availability, usage among tactical populations, and novelty. Each of the devices is embedded with green/red LEDs along with photodiodes to obtain physiological signals via PPG. Each device uses proprietary algorithms to then report RHR in beats per minute (BPM) and HRV in root mean square of successive differences (RMSSD) via smartphone applications.

#### 2.2.3 Manufacturer‐defined data collection methods for RHR and HRV

Each device has different parameters for calculating RHR and HRV, with varying frequencies, requirements, and processes. The Garmin Fenix 6 uses the Elevate V3 optical heart rate sensor to derive heart rate (HR) from photoplethysmography (PPG). However, the manufacturer does not disclose the raw PPG sampling frequency (Hz), rather states that the frequency of collection “varies, and may depend on the level of activity of the user” (Garmin Ltd, 2025b). The device provides processed HR values at a 1‐s interval during activity, but the underlying PPG sampling architecture remains undocumented. The Garmin Fenix 6 measures HR intermittently during sleep and calculates RHR as “the lowest 30 min average in a 24 h period,” (Garmin Ltd, 2019; Garmin Ltd, 2025c). The Garmin Fenix 6 also measures HRV continuously during sleep, separating data into 5‐min windows, then takes the average HRV for the entire sleep period detected by the watch (Firstbeat Technologies Oy, 2019; Garmin Ltd, 2019; Garmin Ltd, 2025a). The Oura Generation 3 ring measures HR at a frequency of 250 Hz (Kryder, 2022). While Oura has not officially disclosed the PPG sampling frequency for the Generation 4 ring, it utilizes a very similar architecture to the Generation 3 device. Both Oura Gen 3 and Gen 4 devices measure HR continuously at night and averages data into 10‐min segments (Oura Health Oy, 2025a). HRV is calculated identically for both Gen 3 and Gen 4 devices by segmenting data into 5‐min samples and averages those for the entire night (Oura Health Oy, 2025b). The Polar Grit X Pro measures HR at a frequency of 1 Hz (Polar Electro Oy, 2024) continuously throughout detected sleep time, though only uses a 4‐h window after sleep onset to calculate RHR and HRV (Polar Electro Oy, n.d.; Polar Electro Oy, 2024). The WHOOP 4.0 wristband measures HR at a frequency of 52 Hz (WHOOP, n.d.) continuously, and reports RHR and HRV using a “dynamic average during sleep… weighted towards your last slow wave sleep stage each night,” (Meserve, 2021; WHOOP, 2024; WHOOP, 2025a). All devices report HRV as RMSSD.

### 2.3 Protocol

Each participant was issued a Polar H10 and instructed on how to properly fit the chest strap according to manufacturer instructions (Polar Electro, 2019). Each night in their own homes, participants would put on the H10 and begin an activity on a separate (non‐worn) Polar watch for data collection. This process ensured that both processed HR (measured at 1000 Hz and reported at 1 Hz in beats per minute) and R to R intervals (measured at 1000 Hz and reported as every heartbeat in milliseconds) were captured. Upon awakening, participants would stop, save, and sync the device to the Polar Flow cloud for further data export and analysis. Subjects were instructed to wear only one device per wrist and to wear the Oura Ring on their finger for at least 30 min before bed and 30 min after waking up, and to synchronize each device with its corresponding smartphone application each morning.

### 2.4 Data processing

Participants that completed at least 10 nights of data collection were included in the study (*n* = 13, 6 females). R to R data (in milliseconds) obtained from the Polar H10 were exported as individual CSV files (one per night) and then processed in Kubios HRV Scientific software (Kuopio, Finland) for RHR and HRV analyses (Rogers et al., 2022; Tarvainen et al., 2014). For each night, HR was visualized, and sleep onset was determined by a noticeable and reliable drop in HR, typically occurring within the first 10–15 min after beginning data collection. For the Polar H10, RHR, and HRV were averaged over the entire night's data. For the Polar RHR and HRV analysis, Kubios “Automatic Noise Detection” setting was enabled to “Medium” and “Beat Correction” was set to Automatic. After processing, data were only included for analysis if <5% of beats were corrected.

All sleep physiological data from wearable devices were synchronized and uploaded to their respective smartphone applications; then manually recorded in a local database. These measures were then directly compared to the corresponding reference values obtained from the Polar H10 and processed in Kubios.

As an additional comparison, nightly RHR and HRV values for each subject were standardized using *Z*‐score normalization. For each participant, the mean and standard deviation of their Polar H10‐derived values across nights within 30 days were used to compute nightly *Z*‐scores for both the reference and wearable device data. This approach allowed for a within‐subject comparison of nightly deviations from baseline, minimizing the influence of interindividual physiological variability. This method was especially important for HRV, which exhibits high interindividual variability, influenced by many factors including age, sex, and fitness level (Laborde et al., 2017; Sundas et al., 2025).

While algorithms determine specifics, each of the tested wearable devices utilizes a combination of accelerometry (i.e., movement) and HR to determine sleep time (Garmin Ltd, n.d.; de Zambotti et al., 2023; Depner et al., 2020; Kryder, 2022; Polar Electro Oy, 2024; WHOOP, 2025b). Per the manufacturer documentation for the Garmin Fenix 6 smartwatch, RHR is calculated as “the lowest 30 min average in a 24 h period,” (Garmin Ltd, 2019; Garmin Ltd, 2025c). Neither the watch nor the accompanying app (Garmin Connect) specifies timestamps of when that 30‐min period occurs. Due to the inability to pinpoint the specific 30‐min period used by Garmin to report RHR, a comparison across devices was not possible; for this reason, the Gramin Fenix 6 was omitted from the RHR analysis. For the Polar Grit X Pro, Polar reports RHR and HRV as an average value only for the first 4 h of sleep (Polar Electro Oy, 2024), therefore the Polar H10 reference values were calculated separately using only the first 4 h for a direct comparison. For Whoop 4.0, HRV is “dynamically weighted” toward the last slow wave sleep of the night, however without knowing this proprietary analysis, the all night HRV average was utilized.

### 2.5 Statistical analysis

To determine agreement, Pearson correlations, Lin's Concordance Correlation Coefficient (CCC) (Lin, 1989) and the mean absolute percentage error (MAPE) were calculated for each metric compared to the criterion measure. For CCC, values <0.80 were regarded as unacceptable, values between 0.80 and 0.89 were poor, between 0.90 and 0.95 were regarded as moderate, 0.95–0.99 as substantial, and >0.99 were regarded as nearly perfect (McBride, 2005). MAPE was calculated as (((Polar H10 − Wearable Device)/Polar H10) × 100), and values >10% were regarded as unacceptable (Chen et al., 2003; Lewis, 1982). Differences in MAPE were calculated by one‐way ANOVA. Pearson's R, CCC, and MAPE were calculated in jamovi (version 2.3) (Jamovi, 2024). Bland–Altman analysis was used to calculate bias and limits of agreement. Mean absolute error (MAE) was calculated as the average absolute difference between the Polar H10 and the wearable device for each night. Differences in MAE were evaluated by a one‐way ANOVA. Post hoc differences in MAPE and MAE were assessed using Tukey's multiple comparisons test, with statistical significance set at *p* < 0.05. All Bland–Altman analysis, ANOVA, and Pearson correlation statistics were performed in GraphPad Prism 10 (GraphPad Software Inc., La Jolla, CA, USA). All data are presented as mean ± standard deviation (SD) unless otherwise noted.

## 3 RESULTS

### 3.1 Participants

Thirteen subjects (male: *n* = 7; female: *n* = 6, age = 33.2 ± 8.6 years) participated in the study.

### 3.2 Resting heart rate

**FIGURE 1:**
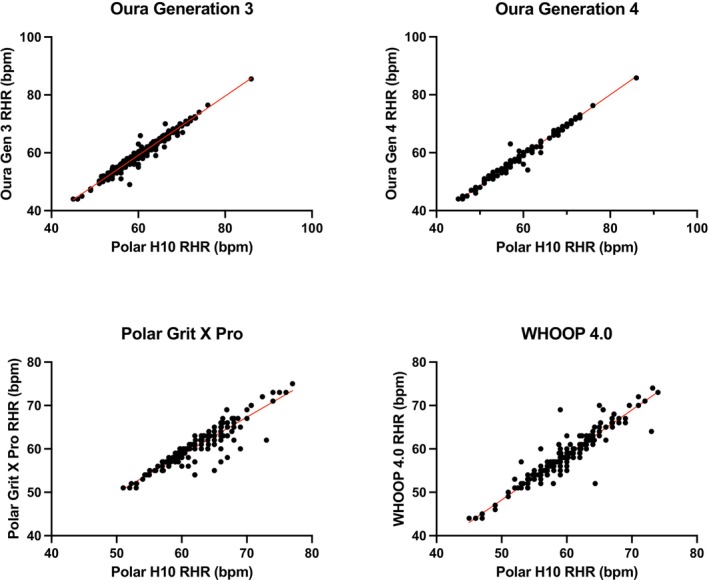
Scatter plots for resting heart rate (RHR) for the Oura Generation 3, Oura Generation 4, Polar Grit X Pro, and WHOOP 4.0. The Garmin Fenix 6 was excluded from RHR analysis.

**FIGURE 2:**
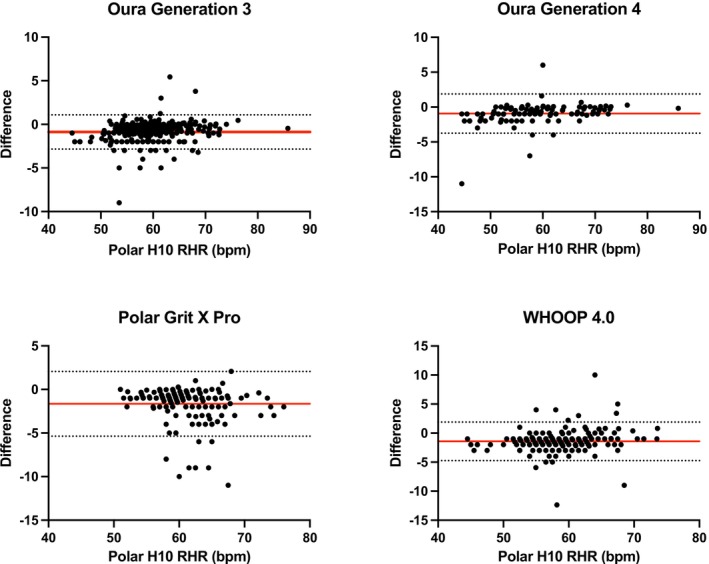
Bland–Altman plots for resting heart rate (RHR) for the Oura Generation 3, Oura Generation 4, Polar Grit X Pro, and WHOOP 4.0. The Garmin Fenix 6 was excluded from RHR analysis.

RHR results are summarized in Table 1. Scatter plots for RHR can be found in Figure 1; Bland–Altman plots can be found in Figure 2.

**TABLE 1:** Resting heart rate accuracy.

Statistic	Oura Gen 3	Oura Gen 4	Polar Grit X Pro	WHOOP 4.0
*N* (number of nights)	470	138	206	288
Mean bias (bpm)	−0.88 ± 1.00^c^, ^d^	−0.94 ± 1.43^c^, ^d^	−0.01 ± 2.13^a^, ^b^, ^d^	−1.41 ± 1.69^a^, ^b^, ^d^
Limits of agreement (bpm)	−2.84, 1.08	−3.75, 1.87	−5.36, 2.06	−4.72, 1.90
Mean absolute error (bpm)	0.98 ± 0.90^c^, ^d^	1.08 ± 1.33^c^, ^d^	1.72 ± 1.30^a^, ^b^	1.78 ± 1.31^a^, ^b^
Pearson's R correlation	0.98	0.98	0.92	0.95
MAPE (%)	1.67 ± 1.54^c^, ^d^	1.94 ± 2.51^d^	2.71 ± 2.75^a^	3.00 ± 2.15^a^, ^b^
CCC	0.97	0.98	0.86	0.91

^a^ Significantly different from Oura Gen 3.

^b^ Significantly different from Oura Gen 4.

^c^ Significantly different from Polar Grit X Pro.

^d^ Significantly different from WHOOP 4.0. Garmin Fenix 6 omitted due to reporting timestamp of RHR calculation, preventing alignment with Polar H10 data.

A one‐way ANOVA revealed significant differences in both mean bias (Fenix 6: highest bias; Oura Gen 3 and Gen 4: lowest bias) and mean absolute percentage error (MAPE) among wearable devices (*p* < 0.01). Post hoc comparisons (Tukey's test) indicated that the Polar Grit X Pro also showed significantly greater bias and MAPE compared to both Oura Generation 3 and Generation 4 (*p* < 0.01 for all comparisons) but was not significantly different from WHOOP 4.0 (*p* > 0.05). The Oura Generation 3 and Generation 4 rings demonstrated similarly low bias and MAPE, with no significant difference between the two (bias: *p* = 0.97; MAPE: *p* = 0.83). Both Oura rings had significantly lower bias and MAPE compared to WHOOP 4.0 (*p* < 0.01).

To account for individual differences in baseline RHR, MAE was also calculated using Z‐score normalized values for each subject. *Z*‐score results for RHR can be found in Table 2. This individualized approach supported the main findings, with both Oura Generation 3 and Generation 4 rings exhibiting the lowest MAE, significantly outperforming Polar Grit X Pro and WHOOP 4.0 (*p* < 0.01). Polar and WHOOP devices demonstrated intermediate accuracy, with MAE values significantly higher than Oura. Lin's CCC aligned with these patterns, with the highest agreement observed for Oura Gen 4 (CCC = 0.96), followed by Oura Gen 3 and Polar (CCC = 0.93), and WHOOP (CCC = 0.87).

**TABLE 2:** *Z*‐score resting heart rate comparisons.

Statistic	Oura Gen 3	Oura Gen 4	Polar Grit X Pro	WHOOP 4.0
Mean absolute error	0.15 ± 0.17^c^, ^d^	0.16 ± 0.24^d^	0.23 ± 0.30^a^, ^d^	0.29 ± 0.37^a^, ^b^
CCC	0.97	0.98	0.92	0.88

^a^ Significantly different from Oura Gen 3.

^b^ Significantly different from Oura Gen 4.

^c^ Significantly different from Polar Grit X Pro.

^d^ Significantly different from WHOOP 4.0. Garmin Fenix 6 omitted due to reporting timestamp of RHR calculation, preventing alignment with Polar H10 data.

### 3.3 Heart rate variability

**FIGURE 3:**
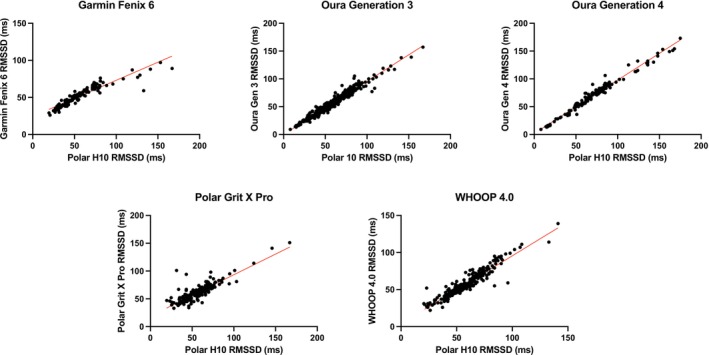
Scatter plots for heart rate variability (HRV) as root square mean for successive differences (RMSSD) for the Garmin Fenix 6, Oura Generation 3, Oura Generation 4, Polar Grit X Pro, and WHOOP 4.0.

**FIGURE 4:**
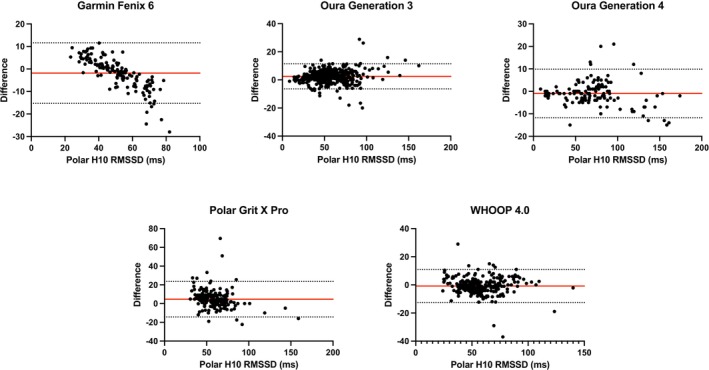
Bland–Altman plots for heart rate variability (HRV) the Garmin Fenix 6, Oura Generation 3, Oura Generation 4, Polar Grit X Pro, and WHOOP 4.0.

HRV results are summarized in Table 3. Scatter plots for HRV can be found in Figure 3; Bland–Altman plots can be found in Figure 4.

**TABLE 3:** Heart rate variability accuracy.

Statistic	Garmin Fenix 6	Oura Gen 3	Oura Gen 4	Polar Grit X Pro	WHOOP 4.0
*N* (number of nights)	150	470	139	206	289
Mean bias (ms)	−1.84 ± 6.86	−2.50 ± 4.56	−0.96 ± 5.52	−4.65 ± 9.67	−0.78 ± 5.98
Limits of agreement (ms)	−15.218, 11.60	−11.43, 6.43	−11.78, 9.85	−14.30, 23.60	−12.50, 10.94
Mean absolute error (ms)	5.29 ± 4.72^b^, ^d^	3.91 ± 3.40^a^, ^c^	3.93 ± 3.98^d^	7.27 ± 7.88^a^, ^b^, ^c^, ^d^	4.17 ± 4.33^d^
Pearson's R correlation	0.96	0.97	0.99	0.86	0.96
MAPE (%)	10.52 ± 8.63^b^, ^c^, ^d^	7.15 ± 5.48^a^, ^d^	5.96 ± 5.12^a^, ^d^	16.32 ± 24.39^a^, ^b^, ^c^, ^e^	8.17 ± 10.49^d^
CCC	0.87	0.97	0.99	0.82	0.94

^a^ Significantly different from Garmin Fenix 6.

^b^ Significantly different than Oura Gen 3.

^c^ Significantly different from Oura Gen 4.

^d^ Significantly different from Polar Grit X Pro.

^e^ Significantly different from WHOOP 4.0.

A one‐way ANOVA indicated significant differences among devices for both mean bias and mean absolute percentage error (MAPE; *p* < 0.01). Post hoc comparisons (Tukey's test) revealed that the Polar Grit X Pro exhibited significantly greater mean bias and higher MAPE compared to all other devices (*p* < 0.01). Garmin Fenix 6 showed significantly higher MAPE than Oura Generation 3 (*p* = 0.0256), Oura Generation 4 (*p* = 0.0133), and WHOOP 4.0 (*p* < 0.0001). There were no significant differences between Oura Generation 3, Oura Generation 4, and WHOOP 4.0 in MAPE (*p* > 0.05).

Regarding MAE, the Polar Grit X Pro demonstrated significantly greater error compared to Garmin Fenix 6, Oura Generation 3, Oura Generation 4, and WHOOP 4.0 (all *p* < 0.01). The Garmin Fenix 6 also exhibited significantly greater MAE compared to both Oura Generation 3 and Generation 4 (*p* < 0.05) but was not significantly different from WHOOP 4.0 (*p* > 0.05). Both Oura Generation 3 and Generation 4 devices showed similarly low absolute errors, without significant difference between them (*p* > 0.99).

When accounting for individual differences by using *Z*‐score normalized MAE values, similar patterns emerged. *Z*‐score results for HRV can be found in Table 4. Polar Grit X Pro and Garmin Fenix 6 showed significantly higher normalized MAE than Oura Generation 3 and Generation 4 rings (all *p* < 0.01). WHOOP 4.0 demonstrated intermediate accuracy with significantly lower error than Garmin Fenix 6 and Polar Grit X Pro (*p* < 0.01), but significantly higher than both Oura rings (*p* < 0.01). Lin's CCC indicated highest agreement with the reference for Oura Generation 4 (CCC = 0.91), followed by Oura Generation 3 (CCC = 0.84), WHOOP 4.0 (CCC = 0.76), Polar Grit X Pro (CCC = 0.79), and Garmin Fenix 6 (CCC = 0.77).

**TABLE 4:** *Z*‐score heart rate variability comparisons.

Statistic	Garmin Fenix 6	Oura Gen 3	Oura Gen 4	Polar Grit X Pro	WHOOP 4.0
Mean absolute error	0.51 ± 0.35^b^, ^c^, ^d^, ^e^	0.22 ± 0.23^a^, ^d^, ^e^	0.27 ± 0.31^a^, ^d^	0.40 ± 0.48^a^, ^b^, ^c^	0.32 ± 0.35^a^, ^b^
CCC	0.77	0.94	0.91	0.79	0.86

^a^ Significantly different from Garmin Fenix 6.

^b^ Significantly different than Oura Gen 3.

^c^ Significantly different from Oura Gen 4.

^d^ Significantly different from Polar Grit X Pro.

^e^ Significantly different from WHOOP 4.0.

## 4 DISCUSSION

The present study evaluated the agreement between nocturnal PPG measurements of RHR and HRV by wearable devices (Oura Generation 3, Oura Generation 4, Polar Grit X Pro, Garmin Fenix 6, and WHOOP 4.0) and a criterion assessment via single‐lead ECG chest strap (Polar H10). Overall, agreement ranged from poor to substantial across tested devices, with the strongest accuracy consistently observed for Oura Generation 4 and Generation 3 rings, closely followed by WHOOP 4.0.

Resting heart rate (RHR) measurements were acceptable in agreement for both Oura devices, Polar, and Whoop. These findings are similar to other investigations into both Oura (Cao et al., 2022; Henriksen et al., 2022) and Whoop (Bellenger et al., 2021; Miller et al., 2021) showing accurate nocturnal readings for RHR. Though the present study found statistically significant differences in mean bias, MAE, and MAPE between these devices for RHR, the differences may be clinically negligible. Previous research indicates that clinically meaningful deviation in RHR typically ranges from 5 to 7 bpm or approximately 10% from baseline (Nanchen, 2018; Quer et al., 2020; Radin et al., 2020). The MAE for RHR in the present study ranged from 0.98 to 1.78 bpm, which is well within the ~5 bpm threshold for clinical relevance.

Heart rate variability (HRV) measurements were most accurate and reliable with the Oura Generation 3, Oura Generation 4, and WHOOP 4.0 devices showing the lowest errors and highest agreement levels. This is in agreement with other studies showing similar rates of error, bias, and agreement for Oura and WHOOP devices (Bellenger et al., 2021; Cao et al., 2022; Kinnunen et al., 2020; Miller et al., 2022). For Polar, our findings are similar to investigations into another Polar watch with similar sensors (Polar Ignite) with similar elevated error (MAE: 5.82 ms, MAPE: 8.72%) for HRV measurements (Budig et al., 2022).

One potential contributor to variability in HRV accuracy is the inherent complexity in proprietary algorithms used by wearable companies to derive HRV from PPG signals. While the manufacturers of the devices evaluated in the present study provide some details as to the segmentation of data collection or how some data is weighted, they do not describe how signal artifact is filtered, how signal quality is interpreted, or how interpolation of missing data is conducted. Time and frequency‐domain HRV indices are particularly vulnerable to artifact and missing data, and differences in processing methods across devices likely contribute to inconsistent HRV metrics (Jarrin et al., 2012). These differences in algorithmic methodologies may contribute to variability between devices and complicate comparisons to gold‐standard ECG‐derived values.

A novel approach in the present study was to apply the *Z*‐score normalization to the physiological data, thus allowing a device comparison further standardized to each individual's baseline. This method enhances the potential detection of significant physiological changes, as variation in physiological metrics is highly personalized. *Z*‐score normalization of both RHR and HRV has been used to improve detection of febrile events using wearable sensors (Kasl et al., 2024). The standardization of both the gold‐standard metrics along with the wearable metrics allowed the inherent variability of each device to be directly compared with the inherent variability of the gold‐standard. With the *Z*‐score normalization, both Garmin and Polar devices exhibited elevated MAE compared to the Oura and WHOOP devices. Because *Z*‐score normalization controls for inter‐individual physiological variability, persistent error suggests that the inaccuracy likely stems from methodological factors—such as signal processing, sensor design, or data segmentation—rather than underlying physiological differences.

A key strength of the current investigation is the relatively large sample size in a real‐world, home‐based sleep monitoring protocol, which emulates typical customer use conditions. Unlike laboratory‐based validation studies, this study introduced individual and natural variability in sleep settings (e.g., body position, room temperature, sleep distractions/interruptions) that the wearable devices had to overcome to maintain signal fidelity. Agreement and MAPE were acceptable for all devices analyzed for RHR performance, suggesting that users and clinicians can trust the sleeping HR metrics gathered from these wearable devices. For HRV, agreement and error were best in the Oura and WHOOP devices. Given the increasing integration of HRV into health and wellness applications, the validity of HRV calculations and algorithms has meaningful implications. High‐performing wearable devices tracking HRV may be suitable for trend monitoring, recovery, and potentially personalized health feedback, particularly when used longitudinally.

Another unique device comparison in the present study was the Oura Generation 3 and Generation 4 comparison. There was nearly identical performance across all RHR and HRV metrics, including the *Z*‐score standardization. This consistency suggests that the algorithmic and hardware performance is stable across both products. Researchers, clinicians, and customers can be confident that Oura Generation 3 and Generation 4 devices provide consistent physiological metrics during sleep. This is especially important when using these devices for longitudinal intervention studies or self‐monitoring.

There were several limitations to this study. Notably, this study was conducted in apparently healthy adults, so generalizability to individuals with sleep or cardiovascular disorders should be considered. For example, atrial fibrillation interferes with normal heart rhythms and thus impacts HRV readings (Chen et al., 2006; Mccraty & Shaffer, 2015). 5.1 million Americans had atrial fibrillation in 2010 and 12.1 million are estimated to have it in 2030 (Colilla et al., 2013). Investigations of HR‐based metrics from wearables should be conducted in populations with atrial fibrillation and other cardiovascular conditions to understand whether these metrics are valid in other populations. Additionally, each of these devices utilizes proprietary algorithms, and while some offer some insight into how their algorithms are designed or weighted, there is little transparency into what metrics affect each device's own “Readiness” or “Recovery Score,” which is typically what is presented to the end user. These algorithms may also be updated periodically, altering how RHR or HRV are calculated, and thus frequent evaluation of their validity should continue.

## 5 CONCLUSION

In summary, the present study demonstrated that wearable devices vary in the accuracy of their nocturnal resting heart rate (RHR) and heart rate variability (HRV) measurements when compared to a gold‐standard single‐lead ECG chest strap. For RHR, the Oura Generation 3, Oura Generation 4, Polar Grit X Pro, and WHOOP 4.0 performed acceptably. However, for HRV, the Oura Generation 3 and Generation 4 rings displayed the highest agreement with the reference, followed by intermediate accuracy for the WHOOP 4.0 and Polar Grit X Pro, while the Garmin Fenix 6 showed notably lower concordance. These findings underscore the importance of continuous validation as new hardware and software updates are released, particularly given the growing role of wearables in personal health monitoring and clinical research. By illuminating specific device strengths and weaknesses under real‐world sleep conditions, this work provides a critical benchmark for consumers, clinicians, and researchers. Future research in diverse populations, as well as those with underlying cardiovascular or sleep disorders, will further refine the translational value of wearable‐derived physiological metrics.

## FUNDING INFORMATION

This study was financially supported by the Air Force Research Laboratory (AFRL).

## CONFLICT OF INTEREST STATEMENT

The authors declare that they have no competing interests.

## ETHICS STATEMENT

The present study was approved as Non‐Human Subjects Research by the Institutional Review Board of the Air Force Research Laboratory (protocol; FWR202200227N) and was compliant with the Declaration of Helsinki guidelines (World Medical Association, 2013).

## Data Availability

The datasets used and analyzed during the current study are available from the corresponding author upon reasonable request.
